# Opinion: Multi-Mycotoxin Reference Materials

**DOI:** 10.3390/foods11172544

**Published:** 2022-08-23

**Authors:** Kai Zhang, Melissa Phillips

**Affiliations:** 1 Food and Drug Administration, Center for Food Safety and Applied Nutrition, 5001 Campus Drive, College Park, MD 20747, USA; 2 National Institute of Standards and Technology, 100 Bureau Drive, Gaithersburg, MD 20899, USA

**Keywords:** multi-mycotoxin reference materials, FDA, NIST

## Abstract

The analysis of mycotoxins in food and feed using liquid chromatography coupled with mass spectrometry is considered advantageous because the hyphenated technology enables simultaneous determination of multiple mycotoxins. Multi-mycotoxin analysis requires special consideration of quality control parameters to ensure proper evaluation of data quality for all target mycotoxins in method development and routine sample analysis. Mycotoxin matrix reference materials, especially certified reference materials, are stable and homogeneous matrices with certified traceability, concentrations, and uncertainty for mycotoxin(s) of interest. The use of these reference materials for single mycotoxin analysis has been a well-accepted practice and should be extended to multi-mycotoxin analysis. This opinion piece discusses the following essential metrological and operational components to improve data quality: (1) purposes of multi-mycotoxin reference materials; (2) comparison of reference materials, certified reference materials, and in-house quality control materials; (3) advantages of using reference materials for multi-mycotoxin analysis; (4) current trends and challenges of multi-mycotoxin reference materials. Potential applications of reference materials discussed here can improve routine mycotoxin determination and will lead to better accuracy and consistency of results. Quality control processes that incorporate reference materials in the field of mycotoxin analysis ensure successful development and implementation of liquid chromatography mass spectrometry-based multi-mycotoxin methods.

## 1. Introduction

Though the history of mycotoxin research is long [1], mass spectrometry (MS)-based mycotoxin analysis was used in the 1980s and 1990s primarily as a complementary tool to mycotoxin quantitation or confirmation using thin-layer chromatography (TLC), enzyme-linked immunoassay (ELISA), and liquid chromatography-fluorescence/UV detector (LC-FLD/UV) [2,3]. Since the early 2000s, liquid chromatography coupled with mass spectrometry (LC–MS) has been increasingly used by mycotoxin testing laboratories [4,5]. Compared to other existing analytical technologies, modern LC–MS offers superior data acquisition speed, sensitivity, and specificity. Multiple mycotoxins can be identified and quantified in one analysis, simplifying sample preparation and increasing throughput. It is not surprising that in this short time, LC–MS has become an indispensable tool for mycotoxin analysis [6,7,8]. While LC–MS has considerable application potential, monitoring and demonstrating the performance of multi-mycotoxin methods in a practical manner is a challenging quality assurance (QA) and quality control (QC) issue.

## 2. Purpose of Multi-Mycotoxin Reference Materials

**Figure 1:**
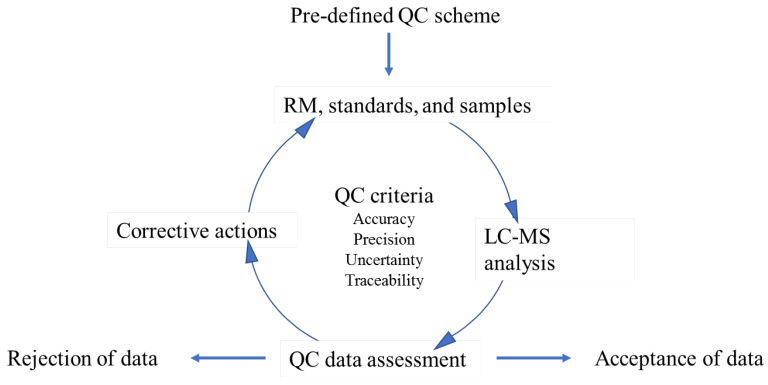
The role of QC samples in LC–MS-based mycotoxin analysis.

Traditionally, to ensure data quality, laboratories have used reference materials (RMs), certified reference materials (CRMs), and/or in-house materials that contain a single mycotoxin or class of mycotoxins as an important QC component during method development and routine sample analysis [9]. Figure 1 illustrates the role of these QC samples in LC–MS-based mycotoxin analysis. Based on pre-designed quality system protocols, suitable QC samples are selected prior to the analysis, and then analyzed with calibration standards and samples using the same analytical methods. The assessment of the control sample data is then used to support decision-making based on data quality (e.g., traceability, accuracy, and precision). If the QC samples are consistently measured within a defined timeframe, the results could be used for short- and long-term statistical assessments (e.g., control chart) [10,11]. Limits of acceptable, warning, and unacceptable values could be statistically established and used for real-time assessment of method performance, which would provide key information to decision makers to accept or reject generated data and take corrective actions to address systemic bias or random errors. Furthermore, as mycotoxin analysis continues to capture global interest [12,13], the establishment of traceability using appropriate QC samples can serve as a metrological component that contributes to international confidence, comparability, and acceptance of measurements [14,15].

## 3. Comparison of Reference Materials, Certified Reference Materials, and in-House Quality Control Materials

To fulfill QC requirements, it is imperative for laboratories to select and analyze suitable control samples, providing actionable information to support pre-defined QC schemes, post-analysis assessment, and the decision-making process. Materials prepared in-house (e.g., contaminated test batches and target analytes spiked into blank matrices) are often used as QC samples. While such practice is flexible, convenient, and economic, uncontrollable errors could be introduced that compromise the quality of QC samples if in-house materials are insufficiently characterized. Finding a balance between operational costs and characterization of in-house prepared samples without compromising accuracy or traceability is challenging. To prepare and characterize multi-mycotoxin QC samples, laboratories also need to pay careful attention to technical challenges in the material planning stage. For example, to save time and control operating cost, labs are rarely willing to establish traceability or characterize sample homogeneity for in-house quality control materials. Instead, spike samples are often used. Spiking target mycotoxin(s) onto a matrix as a positive control seems practical for single mycotoxin analysis but the selection of the matrix and spiking concentrations is not easily extended to multiple mycotoxins. Many food and feed matrices are prone to multi-mycotoxin contamination, making “blank” matrices difficult to find. Spiking-incurred matrices require that the matrix be screened for target mycotoxins first; otherwise, appropriate spiking concentrations for all target mycotoxins cannot be calculated. Even if the concentration of each mycotoxin could be appropriately determined in a selected matrix, individual spiking of multiple mycotoxins would be a tedious operation. Furthermore, each spiking step could introduce unique errors, making the uncertainty related to the QC sample uncontrollable and impossible to estimate. Using multiple single-mycotoxin QC samples might circumvent the above technical barriers, but this approach will slow down operation and, more importantly, for multi-mycotoxin analysis, lead to evaluation of target mycotoxin concentrations under different conditions than in the real samples. Frequently, when multiple single mycotoxin samples are used, the matrices that are available do not match up well with those being analyzed in the study. One should not expect that a corn RM would give much indication of method performance in a fatty matrix such as milk or peanut butter. Problems also arise that even by selecting multiple single mycotoxin samples, there are still mycotoxins that will not be covered, and assumptions should not be made regarding their performance with the method. ISO has established protocols [16,17] regarding characterization of candidate materials that could be used for QC, but following these protocols on a regular basis would significantly increase operational costs. In general, the primary concern regarding in-house-prepared QC samples has long been that the characterization of individual properties is not fully conducted and independently confirmed in terms of traceability, uncertainty, long-term stability, availability, and analyte profile.

When results of poorly characterized QC samples fall out of acceptable ranges, the root cause of errors is difficult to identify because the QC sample itself could be a major source of error. Studies have demonstrated that when using in-house materials, laboratories had difficulty generating consistent results or explaining the inconsistent observations among laboratories [18,19,20,21,22]. Large variability in analytical results offers little confidence to users who rely on the data to make important decisions based on the results. This suggests that either in-house QC samples must be fully characterized prior to their use or reference materials should be used for multi-mycotoxin analysis.

For single-mycotoxin analysis, RMs, especially CRMs, have proven to be an appealing and risk-reducing tool [23,24,25,26,27]. The long and successful history of use of CRMs to ensure measurement accuracy suggests the potential benefit for multi-mycotoxin analysis. In accordance with ISO requirements [16,17,28], an RM must be homogenous, stable, and suitable for its intended purpose. Mycotoxin RMs that have been characterized for one or more properties (e.g., target mycotoxin concentration) could be used for quality control, proficiency testing, and/or method validation. A certified mycotoxin reference material is accompanied by a certificate that documents how the concentration of the mycotoxin(s) is certified with established traceability to the International System of Units (SI) as well as the uncertainty with an estimated level of confidence.

**Figure 2:**
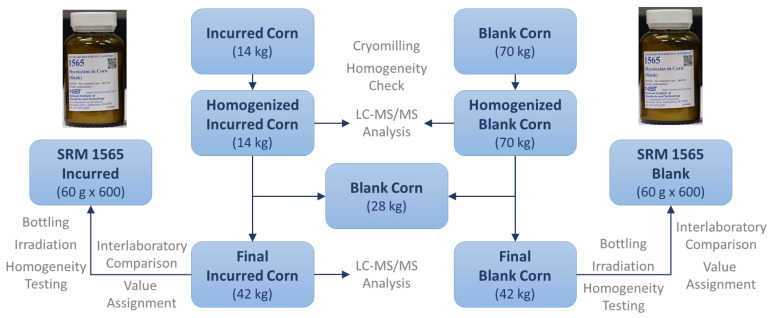
Development of SRM 1565.

**Figure 3:**
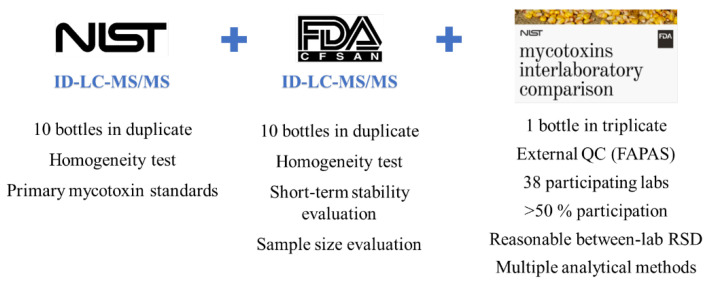
Value assignment of SRM 1565.

A CRM prepared and released by the National Institute of Standards and Technology (NIST) is referred to as a standard reference material (SRM), which was developed following NIST-specified certification criteria [10,29,30]. To determine the identity and assign a concentration of mycotoxin(s) with the highest confidence and metrological traceability in an SRM, a definitive method (e.g., stable isotope dilution-LC–MS) with primary standards was used. Included in the certificate are details of how the mycotoxin SRM was characterized such as certifying laboratories; methods used for certification measurement; how materials were collected, prepared, and homogenized; how results were statistically evaluated; appropriate uses of the SRMs [31,32]. Figure 2 and Figure 3 illustrate how NIST SRM 1565 was developed and the assigned values of the 12 mycotoxins. Apart from these extremely strict requirements and time-consuming preparation and certification, the multi-mycotoxin SRM offers many benefits that in-house materials lack.

## 4. Advantages of Using Reference Materials for Multi-Mycotoxin Analysis

A major benefit of multi-mycotoxin RMs and CRMs is their ease of use. With certified values and uncertainties, CRMs can serve as a real-time indicator for the performance of measurements of multiple mycotoxins in well-characterized and representative matrices. A comparison of results generated by the users to certified values and uncertainties would indicate possible qualitative or quantitative issues and help users to identify the cause of their issues quickly. For example, SRM 1565 was analyzed to assess the performance of an LC–MS-based multi-mycotoxin analysis at FDA/CFSAN (Table 1). The absolute difference between the measured averages and the reference values were calculated and compared to the certified uncertainty (confidence level = 95%, k = 2). For ten out of the twelve mycotoxins, the difference between the respective measurements and reference values was less than the uncertainty defined in the Certificate of Analysis, which suggested satisfactory quantitation in terms of accuracy. For HT-2 toxin, the differences were greater than the defined uncertainty, indicating to the analyst a potential need for corrective action. A common pitfall in quantitative measurements is to ignore the propagation of uncertainty associated with the analytical method and associated reference materials. The uncertainty of the analytical method, while not always available or systemically estimated, could be roughly estimated using the standard deviation of measurements [33]. After factoring in the uncertainty of HT-2 toxin associated with the analytical method, the combined uncertainty (confidence level = 95%; k = 2) was 7.6 ng/g, which was larger than the difference between the certified and the measured values (6.5 ng/g). The measured average of 31.7 ng/g of HT-2 toxin was, therefore, not significantly different from the certified value of 38.2 ng/g. Such analyte-dependent assessment cannot be performed when in-house QC samples are used, due to insufficient uncertainty information. Instead, an arbitrary accuracy or precision threshold is chosen and applied to all target analytes, ignoring the fact that the uncertainty associated with individual analytes varies. It is worth noting that uncertainty varies with the measurement of different mycotoxins in different samples. Applying a pre-defined acceptable threshold to analytical results of in-house materials often creates misleading information regarding method performance of individual mycotoxins. Without a thorough evaluation of uncertainty, or when applying an inappropriate threshold, the threshold could be set too strictly, triggering rejection of good data or unnecessary corrective actions. Conversely, if the threshold is too high, it could result in acceptance of poor data. On the contrary, multi-mycotoxin CRMs provide individual uncertainty of target mycotoxins so that analyte-dependent evaluation can be performed as was demonstrated.

Another benefit of using CRMs is to establish traceability of analytical results to the SI. Traceability is an important requirement for methods used with ISO standards and the use of CRMs can help labs more easily establish traceability. Established traceability of mycotoxin measurements is important to demonstrate the level of competence of testing laboratories [34] and to dictate acceptance of mycotoxin measurements. Levels of mycotoxins detected play an important role in the quality and price of agricultural commodities that are prone to mycotoxin contamination for domestic and international trade. To achieve international acceptance of mycotoxin measurements, traceability of mycotoxin measurements used for both buyers and sellers is recommended [35].

## 5. Current Trends and Challenges

For method development and validation studies, evaluation of accuracy is one of the most important but difficult components. The analysis of RMs and CRMs can demonstrate whether a candidate method could provide accurate quantitation and a related level of confidence. When validation involves multiple laboratories, RMs and CRMs ensure comparability of data and eliminate analytical bias and variability introduced by participating laboratories using individually prepared in-house samples [36,37,38]. One concern with regular use of RMs, especially CRMs, is their cost. Purchasing multiple single-mycotoxin RMs can quickly affect operational costs. Using a multi-mycotoxin RM or CRM is much more cost-effective as one material could provide important QC data for multiple mycotoxins, resulting in a better economy of scale.

The use of multi-mycotoxin RMs and CRMs relieves laboratories from the burden of developing and characterizing in-house QC samples and provides an effective and reliable tool to evaluate mycotoxin measurements in terms of accuracy, precision, and traceability. ISO documents emphasize the importance of RMs, and the prospects for development of new RMs are enormous. However, the application of multi-mycotoxin RMs, especially CRMs, has been stymied by the issue of availability. Though a handful of single-mycotoxin RMs are available, using several single-mycotoxin RMs to evaluate multi-mycotoxin measurements is, as with using single-mycotoxin methods to screen for multiple mycotoxins, an inefficient practice. Thus far, only a few multi-mycotoxin CRMs have been developed due to their lengthy production cycles. To keep up with the increasing need for multi-mycotoxin RMs, international efforts and collaboration to develop various mycotoxin RMs are continuous and ongoing [39]. In recent years, government agencies in the US, EU, Canada, and Brazil have taken the lead in fulfilling the constant need for mycotoxin RMs, especially multi-mycotoxin RMs [40,41,42,43]. The development of multi-mycotoxin RMs involves many target mycotoxins, a long production cycle, and a diversity of candidate matrices. These issues suggest that careful attention must be paid at the planning stage to such matters as the selection of representative matrices and mycotoxins of regulatory and health significance. The development of multi-mycotoxin RMs needs a tremendous amount of resources that no single institute could afford; therefore, a long-term mutualistic collaboration between suppliers and users of RMs should be established to meet existing needs (e.g., multi-mycotoxin RM in animal feeds) and emerging needs (e.g., multi-mycotoxin RM in cannabis) in the foreseeable future.

## Figures and Tables

**Table 1:** LC–MS analysis of three replicates of SRM 1565.

Mycotoxins	Replicates (ng/g)			AVG ± SD (ng/g)	Reference Value ^a^ (ng/g)	Uncertainty ^b^	Combined Uncertainty ^c^	Difference between AVG and RV ^d^
	1	2	3					
Aflatoxin B1	6.9	6.4	9.6	7.6 ± 1.7	7.5	1.7	2.6	0.1
Aflatoxin B2	1.34	1.42	1.76	1.51 ± 0.22	1.43	0.34	0.42	0.08
Aflatoxin G1	0.88	0.87	0.83	0.86 ± 0.03	0.98	0.19	0.19	0.12
Aflatoxin G2	0.82	1.09	0.81	0.91 ± 0.16	0.87	0.24	0.30	0.04
Ochratoxin A	9.6	9.0	9.7	9.4 ± 0.4	9.4	1.2	1.3	0
Fumonisin B1	774	801	788	788 ± 14	805	190	191	17
Fumonisin B2	203	211	209	208 ± 4	217	30	31	9
Fumonisin B3	93.0	91.0	89.0	91.0 ± 2.0	99.3	8.4	8.7	8.3
Deoxynivalenol	427	426	451	435 ± 14	466	69	71	31
HT-2 toxin	29.0	30.0	36.0	31.7 ± 4.0	38.2	6.0	7.6	6.5
T-2 toxin	12.0	13.0	15.0	13.3 ± 1.5	18.4	4.2	4.5	5.1
Zearalenone	67	72	63	67 ± 5	62	31	32	5

^a,,b^ Reference value (RV) and uncertainty are from Ref. [32]; ^c^ Combined uncertainty is calculated following Ref. [33]; ^d^ Calculated as absolute difference between AVG and reference value (RV).

## Data Availability

Data available on request following Public Access to Results of FDA-Funded Scientific Research.
